# A novel mutation *GJA8* NM_005267.5: c.124G > A, p.(E42K) causing congenital nuclear cataract

**DOI:** 10.1186/s12886-022-02386-y

**Published:** 2022-04-15

**Authors:** Ruru Guo, Dandan Huang, Jian Ji, Wei Liu

**Affiliations:** 1 Tianjin Key Laboratory of Retinal Functions and Diseases, Tianjin Branch of National Clinical Research Center for Ocular Disease, Eye Institute and School of Optometry, Tianjin Medical University Eye Hospital, Nankai District, 251 Fukang Road, Tianjin, 300384 China; 2 Department of Ophthalmology, Taihe Hospital, Hubei University of Medicine, Shiyan, Hubei China; 3 Department of Ophthalmology, University of Groningen, University Medical Center Groningen, Groningen, The Netherlands

**Keywords:** *GJA8*, Congenital cataract, Hemichannel

## Abstract

**Background:**

To identify the genetic mutation of a four-generation autosomal dominant congenital cataract family in China.

**Methods:**

Targeted region sequencing containing 778 genes associated with ocular diseases was performed to screen for the potential mutation, and Sanger sequencing was used to confirm the mutation. The homology model was constructed to identify the protein structural change, several online software were used to predict the mutation impact. CLUSTALW was used to perform multiple sequence alignment from different species.

**Results:**

A novel heterozygous mutation, *GJA8* NM_005267.5: c.124G > A, p.(E42K) was found, which cosegregated with congenital cataract phenotype in this family. Bioinformatics analysis of the mutation showed that the surface potential diagram of proteins changed. Several online programs predicted the mutation was ‘Pathogenic’, ‘Damaging’, ‘Disease causing’ or ‘Deleterious’.

**Conclusions:**

A novel mutation NM_005267.5(*GJA8*):c.124G > A was identified in our study. Our finding can broaden the mutation spectrum of *GJA8*, enrich the phenotype-genotype correlation of congenital cataract and help to better understand the genetic background of congenital cataract.

## Background

Congenital cataract is defined as lens opacity that presents at birth or during the first decade of life [1]. It is the leading cause of visual impairment and reversible blindness in childhood [2]. The prevalence of congenital cataract ranges from 0.63 to 9.74 per 10 000, which varies with regional socioeconomic development [3, 4]. Although multiple factors can cause congenital cataract, genetic inheritance is the most common one [1]. Nearly one third of cases have a genetic basis, and the most frequent mode of inheritance is autosomal dominant transmission with a high degree of penetrance [5, 6].

Up to now, at least 100 genes have been identified in syndromic and nonsyndromic congenital cataract [6]. Those known mutant genes encode proteins including crystalline (*CRYAA, CRYAB, CRYBA1/A3, CRYBA4, CRYBB1, CRYBB2, CRYGC, CRYGD* and *CRYGS*), gap junction proteins (*GJA3* and *GJA8*, also called *Cx50*), membrane protein (*MIP/AQP0*), beaded filament proteins (*BFSP1* and *BFSP2*), growth and transcription factors (*HSF4* and *PITX3*), and others (*CHMP4B* and *EPHA2*) [7, 8]. However, there is no clear correlation between genotype and phenotype for inherited cataract. Mutations in the same gene can result in different cataract phenotypes and mutations in different genes can lead to similar cataract phenotypes.

In the present study, we tried to identify the genetic mutation in an autosomal dominant inherited four-generation cataract family. By targeted region sequencing, a heterozygous missense mutation, *GJA8* NM_005267.5: c.124G > A, p.(E42K) was found. This is a novel mutation that has not been reported previously.

## Methods

### Patients

A four-generation Chinese family from Tianjin was recruited in the present study. This family consists of 19 individuals, in which 3 male and 4 female are suffering from congenital cataract. All the patients of this family accepted ophthalmologic examinations and all the patients except the proband had already accepted cataract extraction and intraocular lens implantation surgery. The informed consent was obtained from all subjects of this pedigree after explanation of the nature and possible consequences of the study. This study was approved by the ethics committee of Tianjin Medical University Eye Hospital and followed the tenets of the Declaration of Helsinki. The peripheral blood samples were obtained from ten family members, including five patients and five unaffected individuals, and drawn into an ethylenediamine tetraacetic acid (EDTA) sample tube for further analysis.

### DNA sequencing

The method of targeted region sequencing and data analysis was described in detail before [9, 10]. Briefly, genomic DNA was extracted according to the manufacturer’s standard procedure (MagPure Buffy Coat DNA Midi KF Kit, Magen, China). The qualified genomic DNA was sequenced with PE100 + 100 on MGISEQ-2000. The BGI MGIEasy V4 chip, containing 778 genes associated with ocular diseases through OMIM (Table 1), was used to capture the targeted sequences. Sanger sequencing was used to validate all mutations and potential pathogenic variants after PCR amplification using the primers: forward, GTGCACATTGACCGTTCTGG; reverse, CCTCCAGCCGGAACTTCTTA. Segregation analysis was performed in all available family members. The mutation was also blasted in ESP6500, ExAC, GnomAD, GnomAD-EAS, NCBI dbSNP, HapMap, 1000 human genome dataset and database of 100 Chinese healthy adults in order to rule out the possibility of a polymorphism.

**Table 1:**
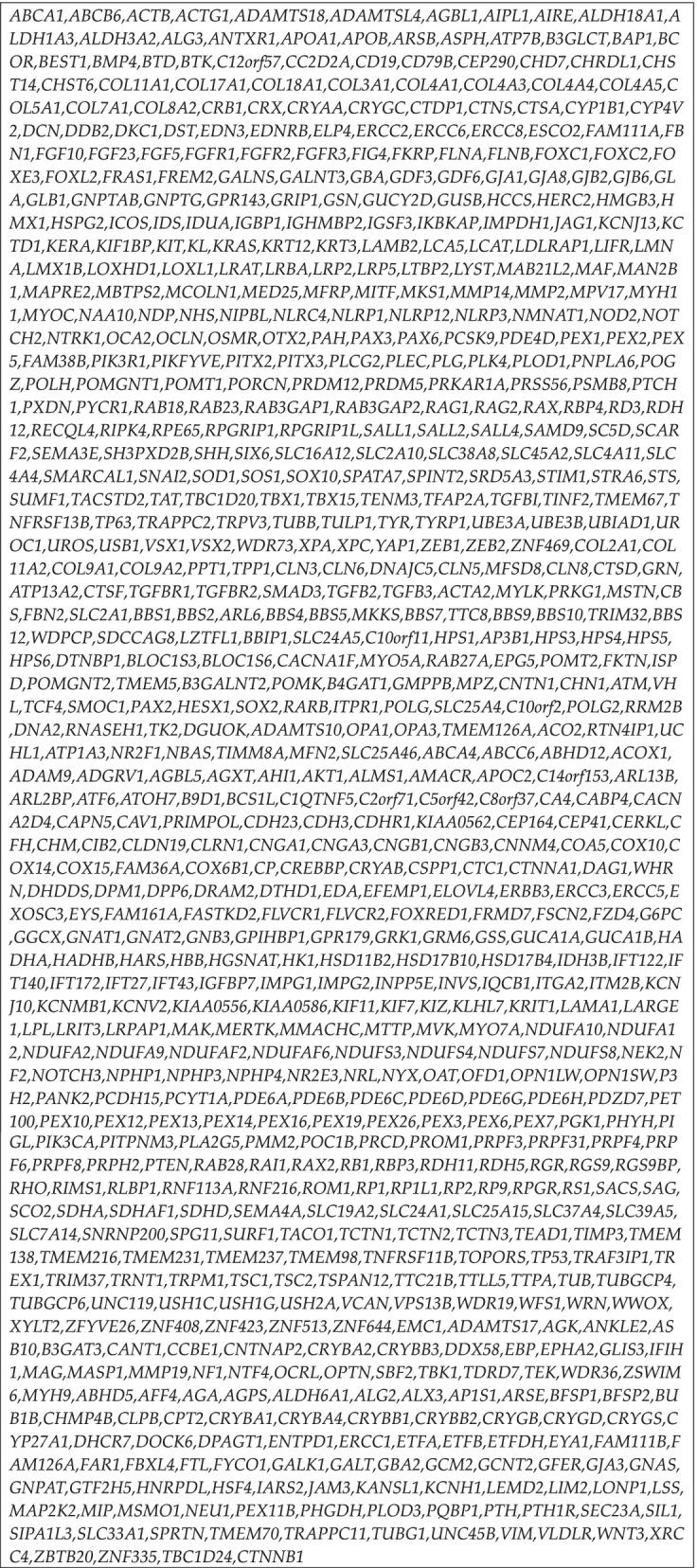
The 778 genes enrolled in our targeted region sequencing

### Bioinformatics analysis

Using the solved structure of gap junction protein alpha 8 as template (Protein Data Bank accession No.6MHY_A), the model structure of homomeric wild and the mutant *GJA8* were constructed by Swiss Model Server (https://swissmodel.expasy.org) and shown with the PyMOL Molecular Graphic system (Delano Scientific). Meanwhile, we used online programs to predict the possible functional impact of the amino acid change, including BayesDel addAF, BayesDel no AF, DANN, DEOGEN2, EIGEN, FATHMM, LRT, MVP, MetaLR, MetaSVM, MutPred, MutationTaster, PROVEAN, PrimateAI, REVEL, SIFT, etc. (http://varsome.com). In addition, multiple sequence alignment from different species was performed by CLUSTALW (https://www.genome.jp/tools-bin/clustalw).

## Results

### Clinical evaluation

**Fig. 1:**
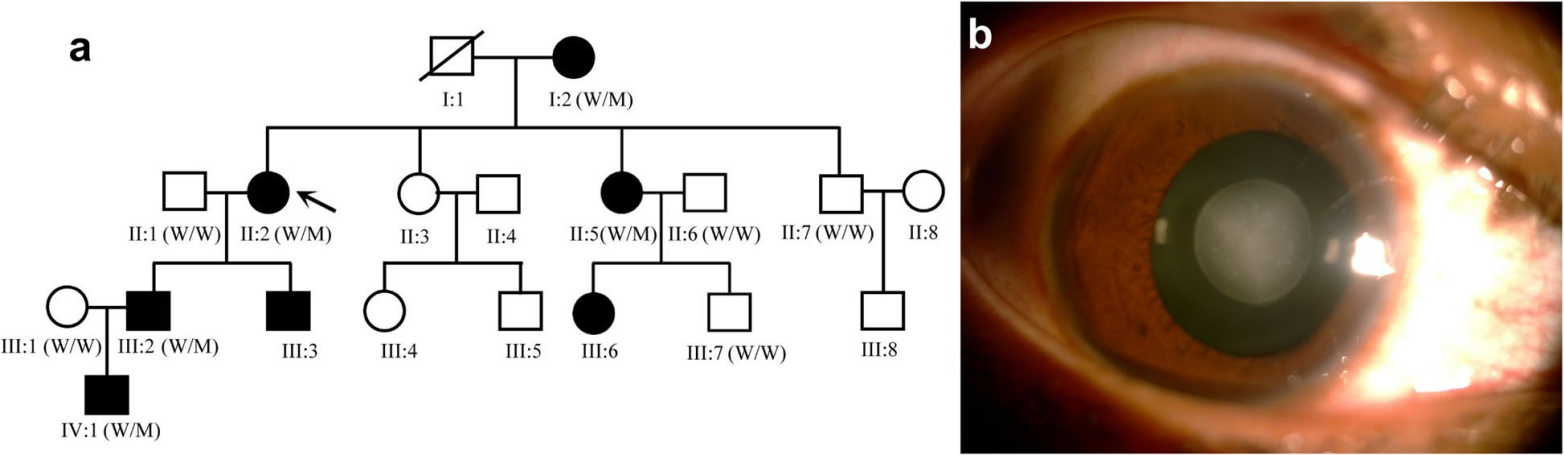
Clinical evaluation of a Chinese pedigree with congenital cataract. **a** A four-generation family with autosomal dominant congenital cataract. The arrow indicates the proband. Squares and circles symbolize males and females, respectively. Black and white denotes affected and unaffected individuals, respectively. W represents wild-type *GJA8* allele; M represents allele with mutation. **b** Slit-lamp photograph of the proband showed pulverulent nuclear cataract

Ten individuals from this four-generation family were recruited in this study, including 5 affected individuals (I:2, II:2, II:5, III:2, IV:1) (Fig. 1a). The proband (II:2), a 46-year-old female, was diagnosed bilateral cataract when she was 3 years old. With the visual acuity decreasing gradually, her left eye underwent cataract extraction and intraocular lens implantation surgery when she was 44 years old. The lens of her right eye was observed to be like a “full moon” with central pulverulent opacities (Fig. 1b). According to the history and medical records, all the other affected individuals were diagnosed bilateral cataract since their childhood and accepted cataract surgery before. Apart from cataract, there were no other ocular or systemic abnormalities.

### Identification of *GJA8* mutations

**Fig. 2:**
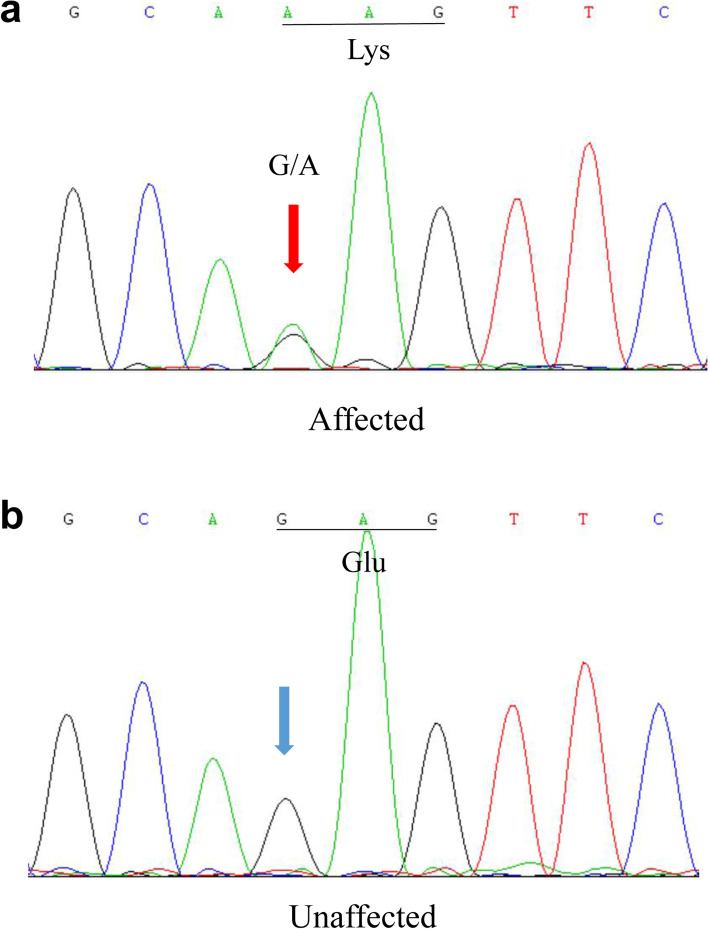
Sanger sequencing of *GJA8*. **a** The red arrow indicates the G>A transition at codon 42 (underlined) in an affected patient from the family. AAG encodes Lys (K). **b** Wild-type sequence from an unaffected family member. GAG encodes Glu (E)

Targeted region sequencing containing 778 genes associated with ocular diseases was performed for the proband (II:2). The average read depth was 139.02X, the sequence coverage of the targeted region was 99.96% and the percentage of the average read depth over 30X was 95.58%. After filtering, one heterozygous mutation *GJA8* NM_005267.5: c.124G > A, p.(E42K) was identified, which was considered to be associated with congenital cataract. This c.124G > A nucleotide replacement causes a substitution of positively charged lysine for a negatively charged wild-type glutamic acid at codon 42 (p.E42K). The mutation was further confirmed by Sanger sequencing (Fig. 2a, b).

**Fig. 3:**
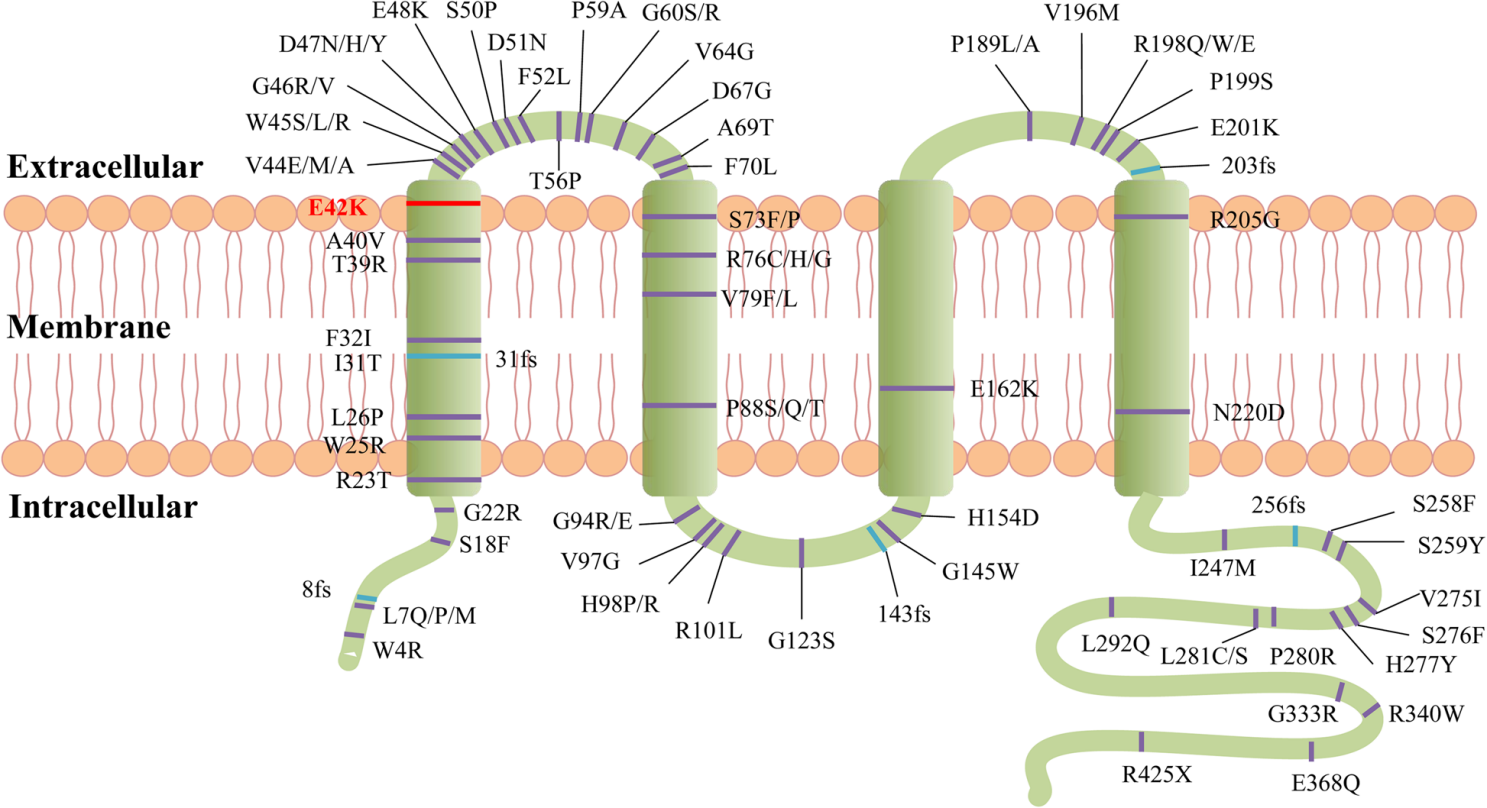
Schematic diagram showing reported human Cx50 mutations (purple bands: missense mutations; blue bands: frame shift (fs) mutations). The red band denotes the mutation p.E42K reported in our study

**Fig. 4:**
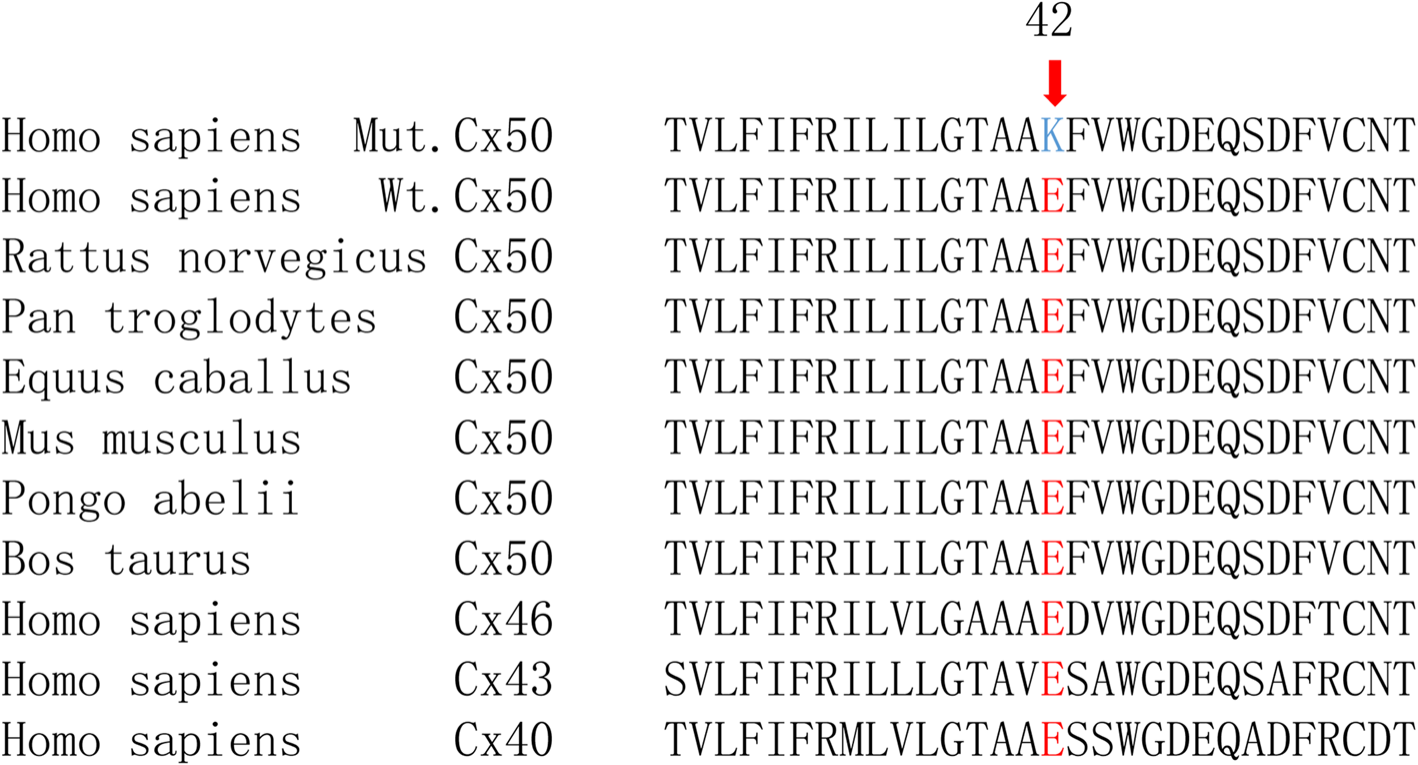
Multiple sequence alignment of the Cx50 amino acid sequence (codons 26-56) from different species, indicating that glutamic acid at position 42 (red) is highly conserved

Segregation analysis demonstrated that this mutation was detected in all the five affected individuals of this family and not detected in the unaffected family members (II:1, II:6, II:7, III:1, III:7), indicating the mutant NM_005267.5(*GJA8*):c.124G > A cosegregated with congenital cataract phenotype in this family. The mutation was not found in ESP6500, ExAC, GnomAD, GnomAD-EAS, NCBI dbSNP, HapMap, 1000 human genome dataset and database of 100 Chinese healthy adults (accessed on February 4, 2022), suggesting the variant may be the pathogenic mutation in this family. The reported mutations in *GJA8* were summarized, and the p.E42K mutant was located within the transmembrane domain of the protein (Fig. 3). According to multiple sequence alignments from various species, glutamic acid at position 42 is highly conserved in *GJA8* (Fig. 4).

### Bioinformatics analysis

**Fig. 5:**
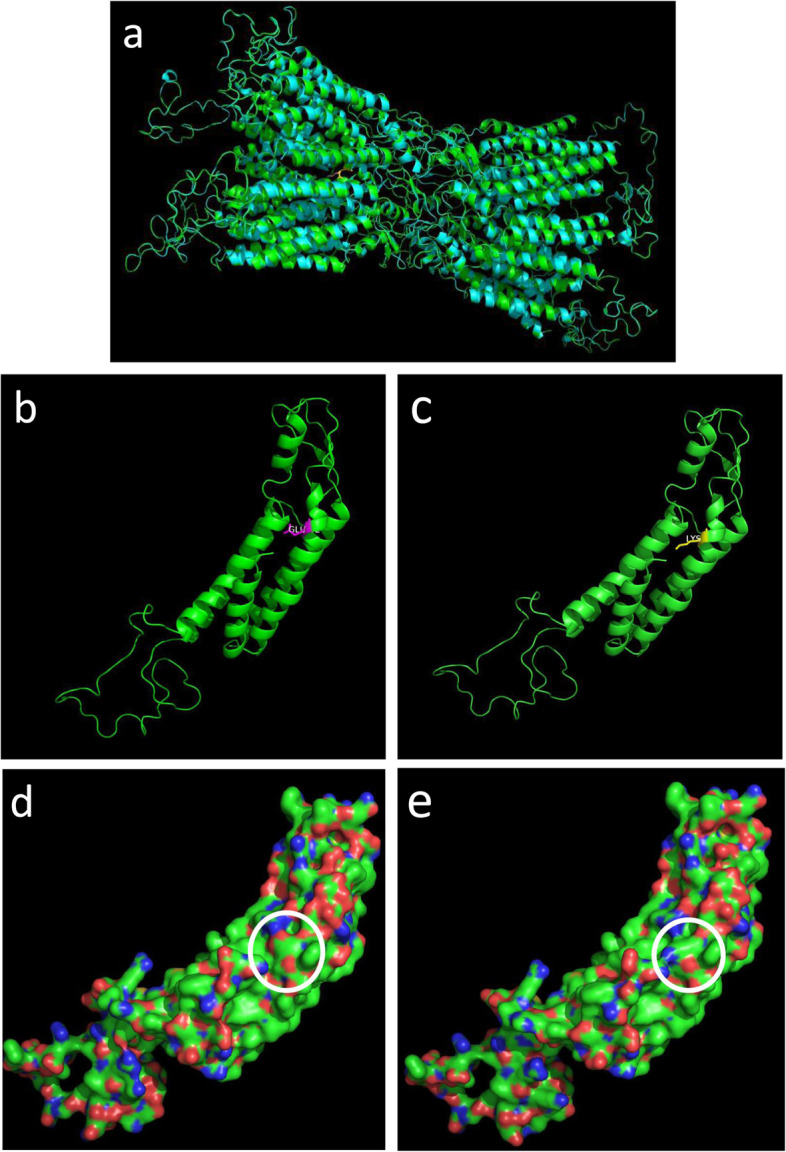
Structure homology modeling of *GJA8* **a** Side view of p.E42K mutant *GJA8* in cartoon form, showing little change on the overall structure of the protein. **b** Local structure of wild-type *GJA8*. **c** Local structure of mutant *GJA8*, had no evident change compared with wild-type (**b**). **d** The surface potential diagram of wild-type *GJA8*, white circle highlights codon 42. **e** The surface potential diagram of mutant *GJA8*, changed significantly compared with wild-type (**d**), the p.E42K mutation caused negatively charged amino acid become positively charged amino acid. Red: negative charge; blue: positive charge

The homology modeling showed overlapped structure of wild and p.E42K mutant Cx50 gap junction channel from the side view and little change was found on the overall structure of the protein (Fig. 3a). The amino acid conformation was also found to have no evident change (Fig. 3b, c). However, the p.E42K mutation caused negatively charged amino acid become positively charged amino acid, and the surface potential diagram of proteins changed (Fig. 5a, b, c, d and e).

Bioinformatics prediction programs were used to assess the functional effects of the p.E42K mutation in Cx50. EIGEN, MVP, MutPred and REVEL got a result of ‘Pathogenic’, MutationTaster got a result of ‘Disease Causing’, LRT got a result of ‘Deleterious’, and BayesDel addAF, BayesDel noAF, DEOGEN2, FATHMM, MetaLR, MetaSVM, PROVEAN, PrimateAI and SIFT showed a result of ‘Damaging’.

## Discussion

In this study, by targeted region sequencing (BGI MGIEasy V4 chip, containing 778 genes associated with ocular diseases through OMIM), we identified a novel heterozygous mutation, NM_005267.5(*GJA8*):c.124G > A, which is responsible for autosomal dominant congenital cataract in a four-generation Chinese family.

The Gap junction protein alpha 8 (*GJA8*) gene, encoding 433 amino acid residues of Cx50, is localized on chromosome 1q21.1 (MIM 600,897, NG_016242.1). Just like other connexins, Cx50 is a transmembrane protein that consists of four hydrophobic transmembrane domains (TM1, TM2, TM3, TM4), two conserved extracellular loops (ECL1, ECL2), and three intracellular regions (a cytoplasmic loop, the NH2 and COOH terminal) [11]. Mutations in *GJA8* have been known to be associated with congenital cataract in humans. To date, more than 80 *GJA8* mutations have been detected in inherited cataract pedigrees (https://cat-map.wustl.edu/). Most of the mutations are located in two extracellular loops (ECL1, ECL2) and COOH terminal while few were reported in transmembrane domains of Cx50 protein.

In our study, the substitution of the positively charged lysine for the negatively charged glutamic acid at position 42 lies on the first transmembrane domain (TM1).The transmembrane domains are responsible for span of plasma membrane and formation of the aqueous pore [12]. Then TM1 is proposed to participate in the oligomerization into connexin hemichannels and the correct transportation of the protein into the plasma membrane [13]. It has been identified that charged pore-lining residues lie in the end of TM1, which are involved in the voltage dependence as a part of the voltage sensor of the slow gate in gap junctions. When glutamate at codon 42 was mutated by a lysine, the profile of the electric field across the hemichannel pore could be changed and the voltage dependence of the hemichannel might be increased [14], thus interfering with the formation of functional gap junctions and leading to cataract formation.

According to the results of bioinformatics prediction, the p.E42K mutation was predicted to have damage effects to the function of *GJA8*, which emphasized the functional importance of this site. In Xenopus oocytes, Pal et al. [15] found that even one single mutant subunit in a gap junction could inhibit channel function, and several reports revealed *GJA8* mutations could inhibit hemichannels [10, 16–18]. Connexin hemichannels can protect lens fiber cells against oxidative damage through a unique cell protective mechanism by transporting the extracellular reductant to the intracellular space. When hemichannels are inhibited, the transportation of reductant from extracellular space to intracellular space will be decreased, thus attenuating the protective effect against oxidative stress which will cause lens cells apoptosis and cells death [19, 20]. Collectively, the mutant will inhibit hemichannels activity and reduce cell tolerance to oxidative stress, resulting in protein aggregation, loss of lens cell function and ultimately cataract formation. However, the exact mechanism of our novel *GJA8* mutation causing congenital cataract needs further functional analysis to confirm.

## Conclusions

In conclusion, our current study is the first to report that NM_005267.5(*GJA8*):c.124G > A mutation is associated with congenital cataract. Our finding can broaden the mutation spectrum of *GJA8*, enrich the phenotype-genotype correlation of congenital cataract and help to better understand the genetic background of congenital cataract.

## Data Availability

The data that support the findings of this study are not publicly available due to their containing information that could compromise the privacy of patient but are available from the corresponding author (WL) upon reasonable request.

## Abbreviations

*GJA8*: Gap junction protein alpha-8
TM1: The first transmembrane domain
PolyPhen-2: Polymorphism Phenotyping v2
PROVEAN: Protein Variation Effect Analyzer
